# Structured chemical class definitions and automated matching for chemical ontology evolution

**DOI:** 10.1186/1758-2946-4-S1-P5

**Published:** 2012-05-01

**Authors:** Lian Duan, Janna Hastings, Paula de Matos, Marcus Ennis, Christoph Steinbeck

**Affiliations:** 1 European Bioinformatics Institute, Cambridge, UK; 2 ETH, Zurich, Switzerland; 3 University of Geneva, Switzerland

## 

Ontologies encode the knowledge of human experts in order to allow computers to automate common tasks in a domain. They are hierarchically organised and backed by computational logic which allows automated inferences of the implicit consequences of explicitly stated knowledge. ChEBI is a database and ontology of chemical entities of biological interest [1]. Within the ontology, chemical entities are classified based on shared structural features and also based on their roles and activities in biological systems. For example, the chemical class ‘aminopyridine’ is defined as ‘Compounds containing a pyridine skeleton substituted by one or more amine groups’, while an example of a role based class is ‘antiviral drug’, which groups together chemical entities that are used as antiviral drugs, regardless of their chemical structure. We have developed a novel semi-automated system for creating structure-based chemical class definitions. Our tool allows curators to draw and visually define shared structural features for classes of chemicals, which definitions are then used to automatically detect class membership across the full chemical database. The front end is based on an extended JChemPaint [2] and the Google Web Toolkit, and the back-end on a custom extension of the Chemistry Development Kit [3]. With this tool, it is possible to define chemical classes based on molecular skeletons, substitute groups, arbitrary parts including cycles of arbitrary length, formulae and overall properties, and these features can be combined using nested logical operators. Matching these definitions to candidate structures from the database is accomplished by means of an in-memory matching procedure, validated against the existing manually curated classification in ChEBI, allowing us to iteratively refine both the definitions of classes as well as to evolve the quality of the classification in ChEBI.

## References

[B1] Degtyarenko K de Matos P Ennis M Hastings J Zbinden M McNaught A Alcántara R Darsow M Guedj M Ashburner M ChEBI: a database and ontology for chemical entities of biological interest Nucl Acids Res 2008 36 Suppl. 1 D344 D350 17932057 10.1093/nar/gkm791 PMC2238832

[B2] Steinbeck C Han Y Kuhn S Horlacher O Luttmann E Willighagen E The Chemistry Development Kit (CDK): An Open-Source Java Library for Chemo- and Bioinformatics J Chem Inf Comput Sci 2003 43 493 500 10.1021/ci025584y 12653513 PMC4901983

[B3] Krause S Willighagen E Steinbeck C JChemPaint- Using the Collaborative Forces of the Internet to Develop a Free Editor for 2D Chemical Structures Molecules 2000 5 1 93 98 10.3390/50100093

